# Access to justice: evaluating law, health and human rights programmes in Kenya

**DOI:** 10.7448/IAS.16.3.18726

**Published:** 2013-11-13

**Authors:** Sofia Gruskin, Kelly Safreed-Harmon, Tamar Ezer, Anne Gathumbi, Jonathan Cohen, Patricia Kameri-Mbote

**Affiliations:** 1 Program on Global Health and Human Rights, Institute for Global Health, University of Southern California, Los Angeles, CA; 2 Open Society Foundations, Public Health Program, Law and Health Initiative, New York, NY; 3 Open Society Foundations, Initiative for East Africa, Nairobi, Kenya; 4 Open Society Foundations, Public Health Program, New York, NY; 5 International Environmental Law Research Centre, Strathmore University, Nairobi, Kenya

**Keywords:** HIV, AIDS, human rights, programme evaluation, public health, stigma, discrimination

## Abstract

**Introduction:**

In Kenya, human rights violations have a marked impact on the health of people living with HIV. Integrating legal literacy and legal services into healthcare appears to be an effective strategy to empower vulnerable groups and address underlying determinants of health.

**Methods:**

We carried out an evaluation to collect evidence about the impact of legal empowerment programmes on health and human rights. The evaluation focused on Open Society Foundation-supported legal integration activities at four sites: the Academic Model of Providing Access to Healthcare (AMPATH) facility, where the Legal Aid Centre of Eldoret (LACE) operates, in Eldoret; Kenyatta National Hospital's Gender-based Violence Recovery Centre, which hosts the COVAW legal integration program; and Christian Health Association of Kenya (CHAK) facilities in Mombasa and Naivasha. In consultation with the organizations implementing the programs, we designed a conceptual logic model grounded in human rights principles, identified relevant indicators and then coded structure, process and outcome indicators for the rights-related principles they reflect. The evaluation included a resource assessment questionnaire, a review of program records and routine data, and semi-structured interviews and focus group discussions with clients and service providers. Data were collected in May–August 2010 and April–June 2011.

**Results:**

Clients showed a notable increase in practical knowledge and awareness about how to access legal aid and claim their rights, as well as an enhanced ability to communicate with healthcare providers and to improve their access to healthcare and justice. In turn, providers became more adept at identifying human rights violations and other legal difficulties, which enabled them to give clients basic information about their rights, refer them to legal aid and assist them in accessing needed support. Methodological challenges in evaluating such activities point to the need to strengthen rights-oriented evaluation methods.

**Conclusions:**

Legal empowerment programmes have the potential to promote accountability, reduce stigma and discrimination and contribute to altering unjust structures and systems. Given their apparent value as a health and human rights intervention, particularly for marginalized populations, further rigorous evaluations are called for to support the scale-up of such programmes.

## Introduction

Integrating legal support into health services is an important strategy for enabling people who are socially marginalized to access justice and address human rights violations that undermine their health [1, 2]. It facilitates holistic care and the realization of rights that have significance for underlying determinants of health, such as the right to education, to an adequate standard of living and to protection from violence and discrimination. It is a particularly valuable mechanism for improving access to justice in settings where people are vulnerable because of their gender, age or health condition.

“Legal integration programmes,” as defined here to mean programmes incorporating legal aid, training and representation into existing health services to improve health outcomes and advance human rights, represent a relatively new approach to addressing structural dimensions of health. The earliest examples come from the United States, where “medical legal partnerships” seek to improve the health and well-being of children, the elderly, the poor and immigrants by eliminating barriers to healthcare and addressing environmental factors that impact health [3]. More recently, the Open Society Foundations’ Law and Health Initiative has funded legal integration programmes in Georgia, Kenya, Macedonia, South Africa, Uganda and Ukraine. These programmes serve vulnerable groups such as people affected by HIV, people in need of palliative care, survivors of gender-based violence, sex workers, Roma and people who use drugs.

In the context of the global HIV response, legal empowerment has begun receiving recognition as an important indicator of health enhancement. The Joint UN Programme on HIV/AIDS cites legal empowerment as a key intervention in national HIV responses [4], while the Global Fund to Fight AIDS, Tuberculosis and Malaria has awarded funding to a number of legal empowerment projects to help them expand their reach and attain key health milestones [5, 6]. That said, it is not yet clear how legal support can best be integrated into health services, within and beyond the field of HIV. Likewise, there is not yet consensus on how best to assess the impact of this work on reducing stigma and discrimination, or on improving health outcomes. Given the key role of stigma as a barrier to HIV prevention and treatment [7, 8], investigating the potential for legal integration programmes to counter HIV-related stigma should be a high priority.

This article presents findings from an evaluation of three Open Society-funded legal integration programmes, all administered by Kenyan nongovernmental organizations (NGOs) (Box 1). The Christian Health Association of Kenya (CHAK), a major provider of HIV-related services in Kenya, implements a legal integration programme through 15 of its 76 health centres and hospitals. The Legal Aid Centre of Eldoret (LACE) is based at a single healthcare facility within the Academic Model of Providing Access to Healthcare (AMPATH) network. AMPATH, a partnership between Kenyan and US academic medical centres, established LACE to represent people whose access to justice is otherwise limited, particularly people living with HIV. The Coalition on Violence against Women (COVAW), a Kenyan human rights organization, established its legal integration programme at a post-rape care centre within Kenyatta National Hospital in 2007.

Specifically we explored how the structure and implementation of legal integration programmes can further human rights principles, how such programmes have the potential to contribute to improved health outcomes and how they advance access to judicial and other forms of redress. These findings, which represent one of the first rigorous evaluations of legal empowerment programmes, suggest that legal empowerment might be a critical health and human rights intervention, particularly for marginalized populations.

***Box 1*. Overview of Kenyan legal integration programmes included in the evaluation**

**The Legal Aid Centre of Eldoret (LACE)** was founded in 2008 to represent people whose access to justice is otherwise limited, particularly people living with HIV. LACE is based within one of the centres operated by the Academic Model of Providing Access to Healthcare (AMPATH), a partnership between Kenyan and US academic medical centres. LACE accepts client referrals from AMPATH staff and also serves clients in the Eldoret community at large.

**The Coalition on Violence against Women (COVAW)** is a Kenyan human rights organization working to eradicate all forms of violence against women. COVAW began its legal integration programme in 2007. The first legal integration site was established at the Gender-based Violence Recovery Centre, a post-rape care centre at Kenyatta National Hospital. Services include direct legal aid, referral to other sources of legal aid and training for clients and service providers on human rights, gender-based violence and related topics.

**The Christian Health Association of Kenya (CHAK)** operates 435 health facilities throughout Kenya, including 25 hospitals. CHAK provides a broad range of HIV-related services, and 20 of its hospitals offer comprehensive HIV care and support. CHAK's legal integration programme seeks to empower people living with HIV by integrating legal services and rights awareness at 10 CHAK hospitals, and a scale-up to five additional sites is underway.

## Methods

### Evaluation model

**Figure 1:**
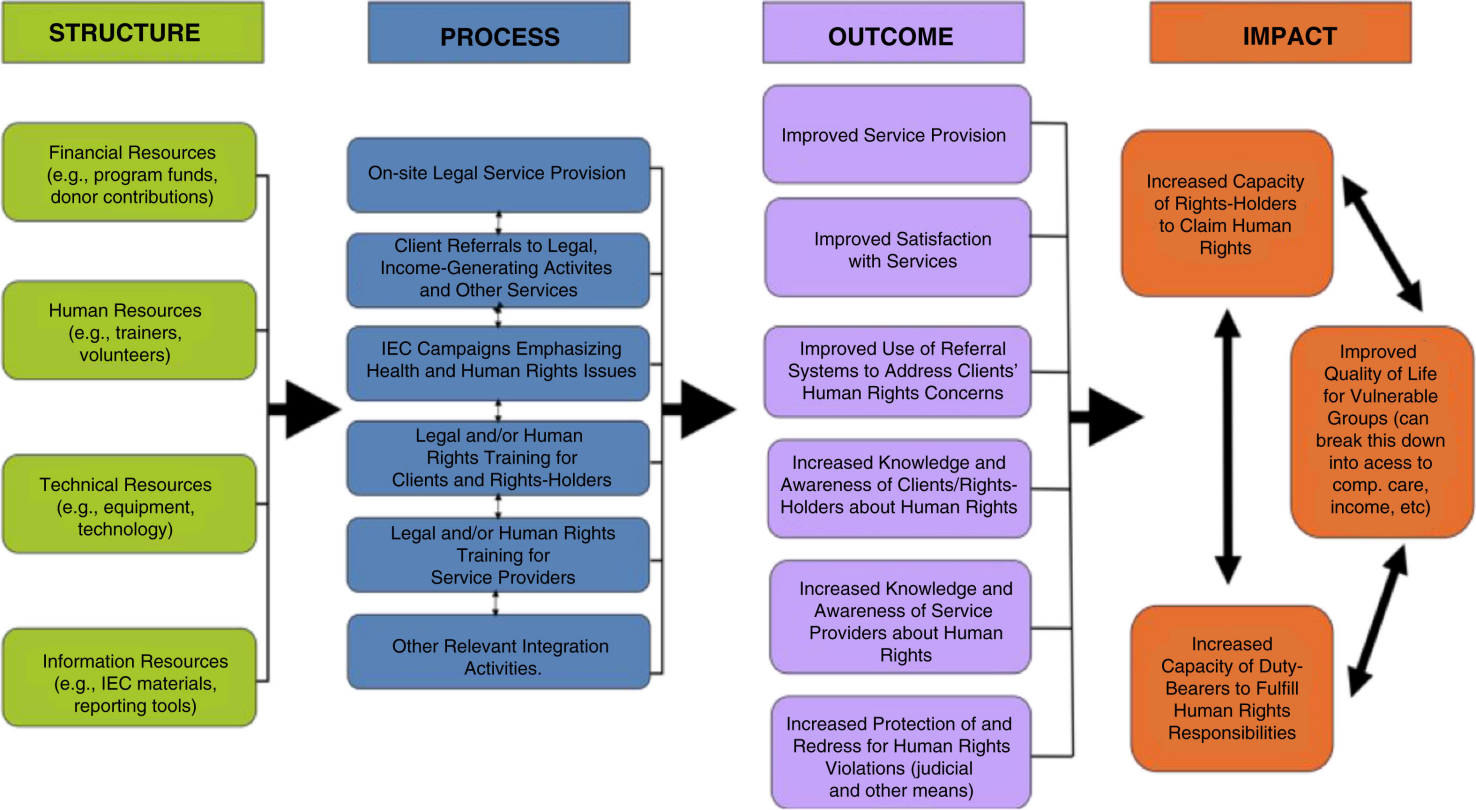
Human rights in logic model development (CARE, CHAK and COVAW's legal integration programmes).

In consultation with the NGOs implementing the programmes, we designed a conceptual logic model to guide efforts to answer the research questions using both quantitative and qualitative methods (Figure 1). The structure components of the model reflect broad categories of resources utilized by the programmes, and the process and outcome components are based on commonly defined activities and objectives. Drawing on the UN Statement of Common Understanding on Human Rights-Based Approaches, we defined long-term impact as building the capacity of rights holders and duty bearers to claim and fulfil rights to improve quality of life for vulnerable groups [9].

A unique feature of the logic model is its attention to the principles of a human rights-based approach, emphasizing participation, accountability, non-discrimination, empowerment and linkage to other rights [9, 10]. We conceptualized the components of the logic model in ways that acknowledged the centrality of specific rights to the work of the programmes – the rights to health, information, education, an adequate standard of living, justice and security of person. In accordance with General Comment 14 of the United Nations Committee on Economic, Social and Cultural Rights, we focused on four key elements of the right to health: the availability, accessibility, acceptability and quality of the goods and services delivered [11].

### Evaluation instruments

The research team identified relevant indicators for all structure, process and outcome components of the logic model, then coded them for the human rights-related principles they reflect. These principles were taken into account during the development of both the quantitative and qualitative evaluation instruments. (The evaluation instruments are described in Supplementary files)

### Data collection

The evaluation focused on Open Society Foundations-supported legal integration activities at the following sites: the AMPATH facility, where LACE operates, in Eldoret; Kenyatta National Hospital's Gender-based Violence Recovery Centre, which hosts the COVAW legal integration programme; and CHAK facilities in Mombasa and Naivasha. The first phase of data collection occurred in May–August 2010, and the second phase occurred in April–June 2011 in order to assess improvements within this time period.

NGO staff independently completed the resource assessment questionnaires, which asked about financial, human, technical and information resources. Quantitative data were captured using existing programme records and routine data. Semi-structured interviews and focus group discussions with clients and service providers were carried out in English or Swahili, depending on which language the person preferred. Swahili transcripts were later translated into English. In some instances, interview and focus group data were collected from both intervention participants and controls. The qualitative case review worksheet was completed by each programme's legal officer, and interviews with the legal officers further contributed to the case reviews.

### Data analysis

Qualitative assessments of resource availability and constraints were made on the basis of resource assessment questionnaires. Quantitative indicators for legal service provision and referral were tabulated according to the records and data provided by the programmes. Interview and focus group discussion transcripts were coded and analysed using qualitative analysis software (NVivo9), with attention to key words, phrases and themes highlighted in the logic model. Case review worksheets were analysed qualitatively, and were further analysed alongside transcripts of qualitative interviews. Findings were grouped according to the evaluation's central areas of inquiry: the nature of the interventions, in terms of the specific training, legal aid and referral activities, and the effects of the interventions, in terms of human rights knowledge and awareness, satisfaction with services and improvements in legal protection and redress.

## Results

Focusing in particular on the process and outcome elements of the logic model, this section highlights key results with potential import to the structure and implementation of legal empowerment programmes more generally.

### Training of clients and providers on legal and human rights issues

Training was a major focus of all three programmes.

LACE reported conducting several trainings on HIV and human rights for clients, most of whom are people living with HIV. The trainings addressed human rights concepts, along with practical legal skills such as writing a will. LACE also trained AMPATH health workers and community health workers in related areas, including child protection.

CHAK client-training efforts capitalized on the existence of well-established support groups made up of and led by people living with HIV. While some trainings were provided directly to support groups, others used a “training of trainers” model to prepare group leaders to educate their fellow group members about human rights. Many also had links to legal aid. For example, a 2009 training for community members, most of whom were people living with HIV, was integrated with a legal clinic at CHAK's Mombasa site. As was generally the case, the training addressed marriage law, succession law, and gender and its impacts on HIV. Immediately following the training, participants had the opportunity to obtain legal information and referrals from on-site lawyers. The training led to at least three cases being carried forward via referrals. CHAK also reported providing human rights training to staff in its HIV, malaria and tuberculosis divisions, again using a “training of trainers” model, where the heads of different clinical departments were then responsible for disseminating information within their departments.

COVAW reported that it regularly carries out legal trainings for Kenyatta National Hospital staff, and for its clients and other community members. Training results were not made available to evaluators as the trainings took place independently of the Open Society Foundations initiative.

### Provision of multiple types of legal aid

The evaluation clearly indicated that the provision of legal representation in formal judicial processes was only one of multiple types of legal empowerment welcomed by clients.

LACE often tried to help clients resolve cases through informal conflict resolution mechanisms. For example, LACE received a complaint from a woman whose husband had deserted her and seven children after learning that she was living with HIV. The husband's family had taken over land that the husband had allocated to his wife. After receiving letters from LACE, the husband's family agreed that the woman could cultivate the land. LACE was later able to persuade the husband to formally transfer the land to his wife's ownership.

LACE's legal aid records document the provision of services to almost 450 clients from the time of the programme's founding in September 2008 through the first half of 2010. LACE reported assisting clients with numerous types of legal documents, including parental responsibility agreements, letters of demand, letters inviting parties for negotiation, birth and death certificates, wills and affidavits. LACE also reported working with clients, prosecutors and the police in criminal cases to facilitate the proper handling of cases.

Most COVAW clients accessed legal services after presenting for medical care. COVAW's legal officer informed clients of legal options such as bringing charges against perpetrators of sexual violence as well as the opportunity to pursue informal conflict resolution. In some cases, the legal officer went on to provide direct legal representation for clients, and in others, COVAW helped clients acquire representation elsewhere. COVAW reported helping clients draft or obtain legal documents relevant to their cases, as well as assisting external paralegals who were working with clients on legal documents.

For evaluation purposes, COVAW was able to provide records for 73 legal aid cases that it handled between January and July 2010. Most clients received only legal information or advice. Two clients additionally received access to informal conflict resolution mechanisms. Eleven clients received referrals to non-legal services, primarily psychological support services. Although only one client received formal legal representation, COVAW was “pursuing” four other cases at the time of the evaluation.

CHAK staff highlighted the value of handling some types of cases at the community level with the assistance of chiefs and other local leaders, most commonly in inheritance and succession cases. For example, CHAK worked with a client who had been unsuccessful in securing the help of her community chief after she was disinherited by her stepsons. CHAK sent a demand letter to the stepsons, copying the letter to the chief, who then became involved in negotiations that resulted in the woman receiving a sufficient financial settlement.

CHAK was unable to provide records of individual legal cases at one of the two sites included in this evaluation, and it had records for only 18 cases at the other site, two-thirds of which occurred in the first half of 2010. Clients in one-third of the cases received access to informal conflict resolution mechanisms. Clients in three of the 18 cases received referrals to non-legal services such as medical services. None of the clients received formal legal representation.

### Referrals

Formal structures for referring clients to legal and non-legal services were found to be crucial to the ability of all three programmes to meet the demand for services. Referrals were made to legal aid organizations, pro bono lawyers, local leaders, government officials and the police. For example, LACE referred clients to the District Labour Officer, District Children's Officer and State Counsel. COVAW commonly sent clients to pre-identified pro bono lawyers for cases requiring litigation. Reflecting the range of services needed for individuals to realize their rights, the programmes’ use of non-legal referrals included referrals to medical services, counselling services, a women's shelter, a “family preservation initiative” and other sources of psychosocial and economic support.

Real and perceived corruption among community leaders, government officials and the police reportedly undermined reliance on referral structures. According to CHAK's legal officer, many clients did not follow through when they were referred to community chiefs because they perceived the chiefs to be either disinterested or corrupt. LACE reported many problems with the police, including cases in which police accused survivors of gender-based violence of giving false evidence and arrested them instead of their perpetrators.

### Knowledge and awareness of human rights and legal issues

Evaluators used focus group data to compare LACE, COVAW and CHAK clients who had received training on legal and human rights issues to control groups of untrained clients. While both sets of clients exhibited general conceptual familiarity with human rights, trained clients appeared to have greater awareness of how and where to access legal services to safeguard their rights. Some trained clients spoke emphatically about how learning about human rights had transformed their outlook or their approach to challenges.

We have been trained on our rights, and we can now confidently talk about our rights before the chief and village elders. (Trained LACE client)

Through [the legal integration program], I know I have basic rights of food, clothing and shelter … We are in hard economic circumstances … and at times we are forced to go to the streets to say that we have a right to food. I'm never afraid to go to the streets because I have been trained on my rights through COVAW. (Trained COVAW client)

[Training] has given us strength and passion to work … We can now educate a person how to live well, be secure and also how to fight for his property. (Trained CHAK client)

Evaluators also used focus group data to compare groups of service providers trained by LACE, COVAW and CHAK to untrained groups. The trained service providers appeared to be better equipped to provide legal and rights-related information and referrals to clients.

From CHAK's training, we came to understand how we can communicate to the patients. Now we have learnt that human rights apply to everybody. Everybody has a right to be treated. We are rolling down that information to the community, and the community is becoming aware of their human rights. (Trained CHAK service provider)

### Service provision and satisfaction with services

In evaluating client and service provider perspectives on the effects of legal integration on service provision and satisfaction with services, comparisons with control groups (people receiving health-related services but not legal training) suggested that legal integration programme clients developed greater access to legal and non-legal resources and felt more empowered. While the benefits of healthcare and social services also supported these feelings, focus group participants identified a clear link with some aspects of the legal services offered by LACE, COVAW and CHAK. They associated the services with, among other issues, being able to secure property, provide financially for loved ones, seek legal redress, speak out against sexual violence, form support groups and engage in advocacy on behalf of others, including people living with HIV.

Service providers who had received training from LACE, COVAW or CHAK reported multiple benefits. They described themselves as being better able to inform clients about their rights, help clients with minor legal matters and refer clients to relevant services.

Service providers’ observations about legal integration contributing to client empowerment corresponded to what clients reported about feeling more empowered as a result of their experiences with all three programmes. For example, trained CHAK providers echoed trained CHAK clients’ views that associated the programme's efforts with clients becoming more comfortable asserting their rights in their interactions with providers.

### Protection and redress for rights violations

The LACE, COVAW and CHAK programmes contributed to improving protection and redress in numerous ways. Health-related human rights issues addressed through casework included discrimination, defamation, land and property ownership, access to housing, probate, debt collection, child maintenance and support, and sexual and gender-based violence. Human rights principles addressed through these efforts included the rights to participation, education, an adequate standard of living, housing and shelter, property ownership, non-discrimination, security of person and justice. In particular, the act of helping clients gain access to legal aid is in itself a means of ensuring the right to justice.

Referrals to non-legal services constituted an important way in which LACE, COVAW and CHAK advanced a range of health-related human rights. For example, CHAK referred a 14-year-old girl who had been sexually assaulted by her stepfather to a CHAK clinic where she could access HIV care and treatment, as well as to a child services organization for psychosocial support. COVAW provided a referral for medical management for a 15-month-old girl who was in her grandmother's custody, as well as referring the grandmother to counselling.

The programmes further sought to systematically improve shared accountability for human rights and shared protection from rights violations. For example, LACE created a network of doctors, nurses, community chiefs and police officers to promote a more effective response to cases of sexual and gender-based violence. LACE's legal officer credited the network with making some chiefs and members of the police more inclined to address rights violations brought to their attention. CHAK established a human rights “watchdog” group in one community. Participants – including the chief, assistant chief, church elders, health workers and trained clients – underwent training to monitor and report human rights violations.

### Obstacles impacting programme effectiveness

Evaluation findings call attention to structural barriers impacting the effectiveness of legal empowerment programmes. Corruption, inaction and mishandling of cases by police were major concerns expressed by informants, particularly in relation to sexual and gender-based violence. Real and perceived corruption and indifference among community chiefs and government officials were other barriers cited, impacting both formal and informal legal action. There were also clients who feared utilizing formal mechanisms because of perceived resource implications or the danger of retaliation from the parties accused of wrongdoing; in some cases, clients received explicit threats.

Another notable obstacle identified through the evaluation was inadequate linkage within health facilities to the legal empowerment programmes. Finally, high staff turnover within the organizations due to high demand across Kenya for staff with legal skills was recognized as a critical barrier. This resulted in the loss of institutional knowledge, undermined programme continuity and limited the value of staff training. Closely related are the challenges associated with acquiring resources to pay staff adequately and ensure long-term programme sustainability.

## Discussion

To our knowledge, no formal evaluations of the rights and health impacts of legal integration programmes have been published to date. Such efforts are necessary not only to inform programme evaluation but also to provide guidance to those who wish to provide effective services in the future.

Evaluation of the three programmes demonstrates health and rights-related benefits associated with training clients and healthcare providers on legal and human rights issues, providing legal aid to clients and referring clients to legal and non-legal resources. The LACE, COVAW and CHAK programmes appear to make positive contributions to clients’ and providers’ awareness of human rights and legal issues, client empowerment and some aspects of health service provision. The programmes also appear to advance the rights of clients and community members at large, as well as to facilitate access to redress for rights violations.

Evaluation findings also call attention to the importance of legal integration programmes looking beyond the courtroom. As noted, it was often more appropriate for LACE, COVAW and CHAK to address clients’ legal needs without engaging with the formal judicial system. In some cases, clients simply needed help with legal documents. In others, the heavy resource requirements associated with utilizing the judicial system made working through informal channels more practical. There were also cases in which fear of retaliation kept clients from initiating civil or criminal proceedings. In those situations, informal conflict resolution offered an alternate means by which clients still might attain justice.

Informal efforts to resolve legal problems, in fact, stood out as a major strength of the three programmes. While the concept of informal conflict resolution has various meanings, evaluation findings suggest the benefits of two types of engagement: work carried out entirely by the programmes themselves, and work that engages community-based mechanisms such as negotiations involving chiefs and community elders.

Referrals may constitute another important means of providing clients with access to justice. By referring clients to both non-health-related and health-related services, these programmes help to advance clients’ rights to justice, health and other rights, with implications also for addressing the underlying social and economic determinants of health [12].

Taken together, the documented experiences point to the importance of understanding the right to justice and the provision of legal aid as multidimensional concepts that involve numerous rights and encompass important opportunities for intervention outside of the formal judicial system.

At the same time, systemic problems plague this sort of work. Most notably, in Kenya (as in much of the world), the criminal justice system must be strengthened so that perpetrators can be prosecuted more effectively and the legal protection of rights appropriately enforced [13].

The research team encountered multiple challenges in carrying out the evaluation. Our quantitative indicators sought to draw on information that often turned out to be inaccessible or missing from programme records, and qualitative data could supplement this information only to a certain extent. One of the largest information gaps resulted from a lack of available records for all but 18 individual legal cases handled by CHAK.

Control group recruitment proved unexpectedly difficult, ultimately reducing our ability to make meaningful comparisons with intervention groups.

The evaluation was also challenged by differences in how human rights terms and concepts are understood by different people. Some concepts and principles, such as “empowerment,” do not translate directly from English into Swahili, and could only be expressed with synonyms. Issues such as these required the research team to make adjustments during the data collection process and to interpret findings in ways that acknowledged differences in how human rights are understood amongst different constituencies.

Evaluation findings provided anecdotal evidence that legal integration programmes can increase access to and utilization of health services. It was, however, beyond the scope of this evaluation to address what might be considered the ultimate question regarding such programmes: whether they are also associated with measurable improvements in health outcomes. Furthermore, while this evaluation makes a compelling case for legal integration programmes as a broadly effective health and human rights intervention, there are inherent limitations to extrapolating these findings to settings with different social and cultural norms or different legal systems.

Evaluation findings overall suggest several strategies for improving future legal empowerment efforts. Optimizing how such programmes make use of referrals should be a priority. Also, better linkages are needed with health facilities where programmes are located. Evaluation data indicated that social work departments and community health workers functioning under the aegis of larger health facilities often served as first points of contact for people who had experienced rights violations. Concern exists that these first points of contact might be missing opportunities to refer clients to legal empowerment programmes.

The cultivation of networks and watchdog groups that involve duty bearers such as the police in their activities stands out as a promising strategy for improving accountability, especially in settings where real and perceived corruption and indifference to rights-related claims deter people from pursuing justice. Community chiefs potentially have a great deal to contribute to the effectiveness of informal conflict resolution, as their role places them at the intersection of formal and informal structures of power: they are simultaneously government officers and perceived upholders of community traditions and standards [14].

The results of this evaluation also have implications for the future monitoring and evaluation of legal empowerment programmes. Findings point to the need for additional rigorous evaluations to inform the scale-up of such interventions. Programmes situated within larger health facilities may be able to capture client and case outcome information more effectively if they build their efforts onto monitoring structures already in place at the larger facilities. The feasibility of plausibility evaluations and qualitative methods for evaluating small-scale programmes should be further explored. An important next step will be to develop measures to determine associations between legal empowerment activities and health outcomes, including those related to treatment adherence and other health-seeking behaviours.

## Conclusions

While the right to justice is widely recognized as a core human right, its health-related implications are only now beginning to be systematically addressed. Integrating legal services with health services does more than facilitate access to justice – an important end in itself. By providing recipients of health services with greater access to justice, legal empowerment programmes can combat a wide range of human rights violations that undermine individual and public health. These programmes have the potential to provide marginalized communities with legal and human rights knowledge, as well as with guidance from legal professionals. Such programmes therefore have the potential to promote accountability for human rights violations, to reduce stigma and discrimination and ultimately to contribute to altering unjust structures and systems that hinder people from making informed and autonomous decisions about their health.
